# MIXED-METHODS EVALUATION OF A NOVEL MULTIMODAL COGNITIVE TRAINING “COGMUSE” IN OLDER ADULTS WITH INSOMNIA

**DOI:** 10.1093/geroni/igad104.2207

**Published:** 2023-12-21

**Authors:** Ashley Curtis, Amy Costa, Madison Musich, Chloe Rzeppa, Kevin Zemon, Isaac Schroeder, Charles Sielert, Fang Wang

**Affiliations:** University of South Florida, Tampa, Florida, United States; University of South Florida, Tampa, Florida, United States; University of Missouri-Columbia, Columbia, Missouri, United States; University of Missouri, Columbia, Missouri, United States; University of Missouri - Columbia, Columbia, Missouri, United States; University of Missouri, Columbia, Columbia, Missouri, United States; University of Missouri, Columbia, Missouri, United States; University of Missouri, Columbia, Missouri, United States

## Abstract

Lack of pharmacological agents to improve cognition has prompted research on behavioral interventions, such as cognitive training (CT). CT benefit is inconsistent across non-patient populations. However, our recent work reveals multimodal commercialized CT shows promise for improving cognition and sleep in older adults with insomnia. Evaluation of non-commercialized CT allows for easy implementation of test scenarios/variables, monitoring and iterative optimization. This study: 1) created a novel non-commercialized CT for older adults with insomnia (“COGMUSE”) and 2) conducted usability testing/feature evaluation. COGMUSE is a computerized multimodal CT (Star Snatcher: targets spatial attention, perceptual motor skill, processing speed; Worse for Wire: targets spatial attention, inhibition). Ten older adults with insomnia complaints (Mage=69.3±3.3) completed 20 minutes of COGMUSE and the Game User Experience Satisfaction Scale (GUESS-18; rating 1-strongly disagree-7-strongly agree across game features). Open-ended feedback was solicited. Compiled transcripts were analyzed independently (A.F.C./F.W.) through deductive content analysis. Frequent topics/themes were identified. GUESS-18 average scores ranged from 3.5/7.0 (enjoyment) to 5.9/7.0 (personal gratification) for Star Snatcher, and from 3.1/7.0 (audio aesthetics) to 5.5/7 (play engrossment) for Worse for Wire. Feedback themes were largely positive (Star Snatcher: good graphics/sounds, easy/enjoyable; Worse for Wire: enjoyable, good instructions). Negative themes included quick movement and lackluster graphics/gameplay “story” (Worse for Wire) and lack of error consequences and practice trials (both games). Older adults with insomnia evaluated COGMUSE as easy and enjoyable to use with acceptable aesthetics. Improvement themes largely focused on instructions and navigation. Next steps include updating COGMUSE and evaluating its preliminary efficacy on cognition and sleep.

